# Consensus document on the progression and treatment response criteria in gastroenteropancreatic neuroendocrine tumors

**DOI:** 10.1007/s12094-018-1881-9

**Published:** 2018-05-15

**Authors:** X. Merino-Casabiel, J. Aller, J. Arbizu, R. García-Figueiras, C. González, E. Grande, P. Jiménez-Fonseca, M. I. Sevilla, J. Capdevila

**Affiliations:** 1 Radiology Department, Hospital Universitario Vall d’Hebron, Barcelona, Spain; 2 Endocrinology Department, Hospital Universitario Puerta de Hierro de Majadahonda, Madrid, Spain; 3 Nuclear Medicine Department, Clínica Universidad de Navarra, Pamplona, Spain; 4 Radiology Department, Complexo Hospitalario Universitario de Santiago de Compostela, A Coruña, Spain; 5 Radiology Department, Hospital Universitario Puerta de Hierro de Majadahonda, Madrid, Spain; 6 Medical Oncology Department, Hospital Universitario Ramón y Cajal, Madrid, Spain; 7 Medical Oncology Department, Hospital Universitario Central de Asturias, Oviedo, Spain; 8 Medical Oncology Department, Investigación Clínica y Traslacional en Cáncer, Instituto de Investigaciones Biomédicas de Málaga (IBIMA), Hospital Universitario Regional y Virgen de la Victoria de Málaga, Malaga, Spain; 9 Medical Oncology Department and Gastrointestinal and Endocrine Tumor Unit, Hospital Universitario Vall d’Hebron, Vall d’Hebron Institute of Oncology (VHIO), Pg Vall d’Hebron, 119-129, 08035 Barcelona, Spain

**Keywords:** Neuroendocrine tumors, Diagnostic imaging, Nuclear medicine, Radionuclide imaging, Molecular imaging

## Abstract

**Purpose:**

Gastroenteropancreatic neuroendocrine tumors are a heterogeneous group of low incidence neoplasms characterized by a low proliferative activity and slow growth. Their response to targeted therapies is heterogeneous and often does not lead to tumor shrinkage. Thus, evaluation of the therapeutic response should differ from other kind of tumors.

**Methods:**

To answer relevant questions about which techniques are best in the assessment of progression or treatment response a RAND/UCLA-based consensus process was implemented. Relevant clinical questions were listed followed by a systematic search of the literature. The expert panel answered all questions with recommendations, combining available evidence and expert opinion. Recommendations were validated through a questionnaire and a participatory meeting.

**Results:**

Expert recommendations regarding imaging tools for tumor assessment and evaluation of progression were agreed upon. Available imaging techniques were reviewed and recommendations for best patient monitoring practice and the best way to evaluate treatment response were formulated.

**Electronic supplementary material:**

The online version of this article (10.1007/s12094-018-1881-9) contains supplementary material, which is available to authorized users.

## Introduction

Neuroendocrine tumors (NETs) are a heterogeneous group of neoplasms that arise from neuroendocrine cells and in particular, affect the gastroenteropancreatic tissue, therefore, being called gastroenteropancreatic NETs (GEP-NETs) [1]. Along this document, GEP-NETs will only refer to well differentiated tumors.

Although improvements in imaging techniques have led to an increased amount of newly diagnosis of GEP-NETs in the past two decades [1], reported incidence is low (2.5–5/100,000/year), currently accounting for 1–2% of all malign neoplasms [1]. This low incidence, apart from the inexistence of concrete risk factors and the demonstrated slow progression, make physicians encounter serious problems in deciding the best approach for diagnosing, assessing progression and evaluating response to treatment. In fact, techniques selected for imaging might have an impact on the definition of progressive disease, which in turn can influence the treatment strategy.

Medical antitumor treatment for GEP-NETs is mainly based on somatostatin analogs, interferon, chemotherapy, locoregional liver therapies, peptide receptor radionuclide therapy and targeted therapies, which are currently arising [2]. In general, NETs response to different therapies is heterogeneous and not necessarily lead to tumor size shrinkage. This is the case of some targeted agents, such as everolimus and sunitinib, which have demonstrated a significant improvement in progression free survival (PFS) in patients with different GEP-NET subtypes, with little or no effect on tumor volume [3]. As these agents do not lead to tumor shrinkage and GEP-NETs are characterized by a low proliferative activity and slow growth, the evaluation of therapeutic response should differ from that of other kind of tumors and be reexamined in the era of targeted therapies.

Despite the current research and progress in GEP-NETs characterization and treatment, there is still a need for higher specificity in diagnosis and monitoring of patients. In an effort to provide some certainty to routine critical practice in this field a RAND/UCLA-based protocol was used to build a systematic and reproducible process. The evidence currently available concerning concrete clinical questions was collected and when no contrasted evidence was found, the participating experts provided clinical practice and expertise-based criteria. This document summarizes the results of the consensus procedure with the objective of helping professionals assessing GEP-NET progression and treatment response.

## Methods

This document of recommendations is the result of a consensus formal process developed following the methodological manual for the preparation of clinical practice guidelines in the spanish national health [4]. A multidisciplinary team composed of experts in medical oncology, radiology, nuclear medicine and endocrinology participated in the process and the formulation of recommendations. A coordinating committee (CC) with two experts, and a recommendation-formulating group (RFG) including seven experts was formed. A content index and a list of clinical questions were developed during a first meeting (Supplementary Table 1). A non-exhaustive systematic literature search was conducted in medline database. A total of 716 publications were obtained. At the CC’s discretion, 48 publications were included in the literature review (47 prioritized from the search and one suggested by the RFG). After reading them, a document answering each clinical question and including potential recommendations was prepared by the RFG. Next, each RFG member individually evaluated all the recommendations in a questionnaire, without having any kind of communication or exchange of opinions. Recommendations were debated during a structured participatory face-to-face meeting. Recommendations that achieved unanimity (100% agreement) or consensus (≥ 75% agreement) were accepted. At the end of the process, a total of 78 recommendations and conclusions were validated. The most important recommendations were formally categorized with their level of evidence (LE) and recommendation degree (RD), according to the oxford center for evidence-based medicine 2011 levels of evidence.

## Results

### Assessment tools for imaging GEP-NETs

Multiphasic contrast-enhanced abdominal and pelvic computed tomography (CT) or magnetic resonance imaging (MRI) are recommended for all patients with suspected GEP-NETs [5]. It is remarkable the need for multiphasic images to avoid the miss-diagnosis of liver metastasis that can be hidden in early phases of the contrast. MRI is worth highlighting because of its non-ionizing radiation nature, being a choice in patients who require long-term follow-up or screening. However, the most widely used indication for MRI is for focused examinations for problem solving [6]. On the other hand, nuclear medicine techniques are essential to estimate the total disease burden. Currently, the standard for GEP-NETs imaging is somatostatin receptor (SR) scintigraphy (SRS) with ^111^In-DTPA-octreotide, although in the near future this might be replaced by positron emission tomography (PET) using somatostatin receptor (SR-PET), due to its high sensitivity and specificity for evaluating localized NETs in the thorax, bone and abdomen [5]. SR imaging (SRI) is often used for the initial staging of the disease and to evaluate the SR status, and thereby the patient’s eligibility for somatostatin analogues (SSA) therapy [5]. Therefore, morphological imaging by CT or MRI and molecular imaging techniques must be combined for primary tumor detection and staging, as well as for evaluating tumor SR status (agreement: 100%; LE/RD: 5/D). Thus, SRI is the technique of choice for GEP-NETs staging, which should nowadays be performed by PET, when ^68^Gallium-DOTA-TOC/-NOC/-TATE is available (agreement: 88%; LE/RD: 5/D).

### GEP-NETs progression

#### Evaluation of disease progression

In the case of the thorax, abdomen and pelvis, multi-slice intravenous contrast-enhanced CT is the usual technique for evaluating extrahepatic disease (agreement: 100%; LE/RD: 5/D), as recommended in clinical guidelines because of its efficiency and accessibility [6]. MRI is considered to be superior to CT in the detection and follow-up of liver metastases apart from avoiding radiation exposure, being this especially valuable in young patients requiring long-term serial imaging. A combination of diffusion-weighted MRI (DW-MRI) and dynamic contrast-enhanced MRI (DCE-MRI) can improve the specificity and the evaluation of treatment response, and have the potential to demonstrate early physiological changes that may predict responders and non-responders early in the course of treatment [7].

In patient-based analysis, PET/CT shows higher accuracy for lymph node and bone lesions, while in organ-based detection whole body MRI (WB-MRI) shows more accuracy for liver and bone metastases and PET/CT for lymph node and pulmonary lesions [8]. The MRI study should include a combination T1-, T2-weighted and DW imaging, making unnecessary the administration of intravenous contrast (agreement: 100%; LE/RD: 5/D) [8]. When available, SR-PET is generally recommended for evaluating the appearance and/or the progression of GEP-NET metastases, although ^68^Ga-DOTA peptides and WB-MRI can be considered for evaluating bone metastases in patients with spine symptoms (agreement: 100%; LE/RD: 5/D) [9, 10]. SR-PET is useful for staging and evaluating treatment response of grade (G)1 NET patients. As the appropriateness of both SR-PET and ^18^F-FDG-PET in G2 NET patients is widely debated, a multimodality diagnostic approach using both PET tracers could be useful in G2 NET patients. Well-differentiated GEP-NETs (low to intermediate grade) can differ in clinical presentation, SR expression, functionality and proliferation rate when compared to poorly differentiated (high grade) tumors and large or small cell carcinomas. Thus, the choice of the molecular imaging technique to be used depends on the proliferation rate and grade of the disease [11]. Both SRS and SR-PET (^68^Ga-DOTATOC/NOC/TATE) are recommended for following-up well-differentiated GEP-NETs metastases and those expressing SR, being SR-PET preferable when available (agreement: 88%; LE/RD: 4/C). In fact, SR-PET can be recommended to follow-up all well differentiated GEP-NETs and metastases [12] (agreement: 100%; LE/RD: 4/C).

### Patient monitoring

#### Morphological imaging

The moment of progression needs to be defined to decide the best time to change treatment. The expert panel recommends establishing disease progression following RECIST criteria, by an increase of 20% or more in the sum of targeted lesions diameter, compared to the lowest value obtained with the treatment in course (nadir) or the appearance of new lesions. Other considerations for radiologic progression are still under investigation (agreement: 100%; LE/RD: 5/D). In any case, the same type of radiological assessment study should be used for the follow-up of lesions, since the use of methods with different accuracy should lead to an erroneous interpretation of the appearance of new lesions or an increase in previous ones (agreement: 100%; LE/RD: 5/D).

The adequate frequency of follow-up has not been established, but it is necessary to contemplate the need to detect early progression and avoid risks (radiation, use of contrast media, etc.) and costs caused by very frequent follow-up in stable patients. Although prospective studies analyzing benefits and hazards of frequent imaging assessment are lacking, Table 1 shows recommendations regarding the frequency of follow-up evaluation formulated by the RFG.

**Table 1:** Recommendations on follow-up evaluation frequency

Recommendations in the follow-up of metastasic GEP-NET	LA (%)	LE/RD
Follow-up of metastatic G1–2 GEP-NETs should initially be performed every 3 months for 2 years; in case of stable disease, assessment every 6 months is recommended. Once disease progression appears, going back to 3 months follow-up schedule is recommended	100	5/D
Morphological imaging follow-up of G1–2 small intestine GEP-NETs should be carried out every 3–6 months; in case of stable disease, the follow-up schedule could be extended to 6–12 months. Once progressive disease appears going back to 3–6 months follow-up is recommended. In case of clinical or biochemical progression, molecular imaging should be also considered	89	5/D
As GEP-NETs are very heterogeneous and tumors with the same characteristics (primary site and Ki-67 index) may show different clinical evolution, careful follow-up should be initially performed in certain patients.	100	5/D
In the case of G1–2 GEP-NETs showing rapid growth, considering re-biopsy is recommended	100	5/D

*LA* level of agreement, *LE/RD* level of evidence/recommendation degree

#### Molecular imaging

Imaging using radiolabeled SSA is indicated to detect recurrent or progressive disease during the follow-up of patients with known metastatic disease [13]. ^111^In-Pentetreotide is more sensitive than planar or planar plus SPECT when evaluating progression in metastatic GEP-NETs [14]. However, the sensitivity of ^68^Ga-DOTA peptides imaging is higher than ^111^In-Pentetreotide, even when SPECT/CT scans are used [14]. ^18^F-FDOPA PET is also more accurate than ^111^In-Pentetreotide for restaging metastatic GEP-NETs and should be performed when ^68^Ga-DOTA peptides are not available [15] and it is recommended instead of ^68^Ga- DOTA peptides when serotonin is overexpressed [16].

The gain in glucose utilization in GEP-NETs has been found to coincide with a loss of SR expression and the higher the NET grade is, the higher its glucose metabolism [17]. Thus, it has been suggested that ^18^F-FDG PET should be limited to SR-negative NET patients, although G1–2 patients may also initially have ^18^F-FDG-positive tumors and may develop ^18^F-FDG-positive lesions during follow-up [18].

Compared to ^111^In-pentetreotide SPECT/CT, ^68^Ga-DOTATATE PET/CT detects additional lesions in 71.0% of patients and when it is compared with CT scan, additional lesions are frequently detected, having a high management impact in a significant number of patients [19].

High-grade, poorly differentiated NETs often have limited expression of SR [20], which can lead to negative SR imaging results and make the molecular investigation difficult [18]. Adopting a dual-tracer approach, assessing SR expression and glycolytic metabolism could support better individual therapy selection in GEP-NET patients. The combination of ^18^F-FDG and ^68^Ga-DOTATATE PET/CT has a significant impact on the therapeutic decision and is helpful in the individual therapeutic approach [21]. Expert recommendations regarding imaging, ^18^F-FDOPA and ^18^F-FDG, for routine clinical practice if available with SSA are formulated in Table 2.

**Table 2:** Recommendations on imaging with SSA, ^18^F-FDOPA and ^18^F-FDG

Recommendations	LA (%)	LE/RD
Imaging using radiolabeled SSA (^111^In-Pentetreotide, ^68^Ga-DOTA peptides) is recommended in patients with known metastatic disease, to detect recurrence in the follow-up or progression during treatment [13]	100	5/D
Imaging using radiolabeled SSA should be considered when a change in the treatment strategy is anticipated [30]	100	5/D
^68^Ga-DOTA peptides PET/CT may detect residual disease when suspected, despite negative morphological imaging or ^111^In-pentetreotide SPECT/CT results [19]	100	2/B
^18^F-FDOPA PET is preferred over ^111^In-Pentetreotide, and when ^68^Ga-DOTA peptides are not available [15, 19]	100	2/B
Radiolabeled SSA imaging is mandatory before referring patients with extended disease to peptide receptor radionuclide therapy [20]	100	5/D
Performing ^18^F-FDG PET is recommended in G1 and G2 GEP-NETs when radiolabeled SSA imaging is negative and when tumors exhibit a rapid progression on morphological imaging or SSTR PET/CT (over a period of less than 6 months) [18]	100	3/B
The adoption of a dual-tracer approach including imaging using radiolabeled SSA and ^18^F-FDG-PET imaging in GEP-NET patients helps for better characterizing of tumors and may subsequently impact therapeutic decisions [21]	100	2/B

*LA* level of agreement, *LE/RD* level of evidence/recommendation degree

### Assessment of treatment response evaluation

Adequate assessment of response after therapy will not only help predict prognosis, but also influence treatment decisions. Due to low proliferation rate of GEP-NETs, monitoring changes in tumor size is suboptimal to assess the response to systemic therapies, especially since they tend to stabilize the disease rather than to shrink the tumor.

In GEP-NETs patients, tumor response is basically assessed through blood circulating biomarkers and imaging. According to a consensus on biomarkers, imaging was considered the best modality to measure treatment effectiveness. However, no agreement on the optimum imaging method to use in different types of tumor was achieved. CT/MRI in conjunction with SRI was appropriate as a routine measure. Within the nuclear medicine tools, PET/CT with ^68^Ga-labelled SSA or ^18^F-FDOPA was considered the best method for NET imaging in centers of excellence [22]. ^68^Ga-DOTATOC is a useful complement to morphologically oriented imaging methods for early assessment of progressive disease, after finalizing peptide receptor radionuclide therapy, namely in that it detects new and as-yet undiscovered lesions during whole-body PET [23] (agreement: 100%; LE/RD: 2/B). ^18^F-FDG PET should be considered for tumor response assessment, especially when positive in G1 and G2 tumors, since the presence of ^18^F-FDG-positive tumors correlates strongly with a higher risk of progression [18] (agreement: 100%; LE/RD: 2/B). Besides, the panel of experts also suggested using ^18^F-FDOPA-PET for monitoring treatment response, especially in those patients with elevated serotonin and catecholamine pathways tumor markers in urine and plasma, regardless of the type of therapy prescribed [24] (agreement: 78%; LE/RD: 2/B).

The RECIST 1.1 criteria, currently used for treatment response assessment in general oncology, rely on morphological imaging and state that a maximum of two lesions per organ and five in total should be measured [25]. Likewise, RECIST criteria may be difficult to apply in GEP-NET patients with hepatic metastases due to changes in their appearance following contrast administration, as well as the coalescence of lesions and the subsequent inability to delineate individual masses. Well-differentiated GEP-NETs, especially those from pancreatic origin, are highly angiogenic and CT images are often strikingly modified by vascular endothelial growth factor inhibitors in the inner part of the tumor, with only limited size variation, producing hypodensity that is not adequately captured by classical RECIST criteria. Choi criteria, used in gastrointestinal stromal tumors, or modified RECIST criteria (mRECIST), used in hepatocellular carcinoma, may be alternative methods to assess response, albeit their utility in GEP-NETs has not been proven yet. In any case, the expert panel opinion is that new or modified evaluation criteria should report the liver metastatic fraction as a potential measure of response (agreement: 100%; LE/RD: 5/D). Besides, NET response to different types of treatment is heterogeneous and depends on the mechanism of action. Thus, methods evaluating GEP-NET response to therapy must also take these differences into account [2].

### Evaluation of response to multikinase or mTOR inhibitors

The targeted agents everolimus and sunitinib have demonstrated a significant potential improvement in PFS in patients with different GEP-NET subtypes, with little or no effect on tumor volume [2]. Some of the criteria to assess response to targeted therapies have been evaluated: Chun and Choi, new criteria obtained by DCE ultrasonography (DCE-US) and RECIST. Chun criteria have proven being relevant for patients with metastatic colorectal cancer treated with bevacizumab [26]. New criteria obtained by DCE-US warrant further exploration in NETs and comparison to RECIST criteria for therapeutic evaluation. Regarding Choi criteria, preliminary data in patients with pancreatic NETs suggest that they could help identify patients who might benefit from sunitinib or everolimus therapy earlier [27]. Evaluation of tumor response to targeted therapies requires a combined approach assessing both morphological (tumor size and density) and functional tumor changes. Finally, DCE imaging techniques (perfusion CT, DCE-MRI, etc.) should be considered in future studies of antiangiogenic agents [28], but not in the case of mTOR inhibitors, because these do not show significant changes [29].

### Pseudoprogression

Pseudoprogression is a false positive progression, perceived as an increase in the number of metastatic lesions, especially 1 cm or smaller liver lesions shown by contrast-enhanced CT or MRI. This potential pitfall frequently leads to misinterpretation of progressive disease and premature cessation of the current regimen, which can actually remain effective [3]. When pseudoprogression happens, the new lesion tends to further decrease in size and density on the follow-up images. For this reason, the correlation between clinical and biochemical parameters and the overall response in other tumor locations are recommended when an atypical treatment response is expected (agreement: 100%; LE/RD: 5/D). If a response pattern is doubtful or atypical and no immediate implications related to management exist, imaging revaluation in 8–12 weeks should be considered (agreement: 100%; LE/RD: 5/D).

Despite the fact that this consensus was created following a solid, rigorous and recognized methodology for this type of documents, there are a series of limitations that must be considered.

The most obvious limitation is that expert opinion and consensus do not compensate the lack of scientific evidence. However, it is capable of establishing expert recommendations to optimize decision making in different clinical and therapeutic conditions. Another limitation is that, the systematic review of the literature was not exhaustive to focus on the most recent and up-to-date evidence. Finally, it should be noted that the revised topics were chosen by the experts based on their experience.

## Conclusions

GEP-NETs are low incidence heterogeneous tumors with usually indolent progression. This has made it difficult to conduct clinical trials and has made the evidence available for most routine interventions weak. On the other hand, in the last years, there have been several new therapeutic options for patients with GEP-NETs. However, the lack of comparative trials makes difficult to place these drugs in a standard sequence. In this context of uncertainty, treatment option should be discussed in a multidisciplinary board, encompassing professionals from several disciplines such as oncologist, surgical oncologist, endocrinologist, radiologist and nuclear medicine specialists.

With this essence in mind this consensus was created, allowing to develop recommendations that assist clinicians in the decision-making process. To accomplish the previous, available morphological and molecular imaging techniques were reviewed, recommendations for best patient monitoring practice were proposed and the optimal way to evaluate treatment response were formulated.

## Electronic supplementary material

Below is the link to the electronic supplementary material.

Supplementary material 1 (DOCX 18 kb)
