# Supplementary material for: Consensus document on the progression and treatment response criteria in gastroenteropancreatic neuroendocrine tumors
Source: Clin Transl Oncol. 2018 May 15;20(12):1522–8. doi: 10.1007/s12094-018-1881-9 (PMC6223716; doi:10.1007/s12094-018-1881-9)
Supplement: Supplementary file 1 — Supplementary material 1 (DOCX 18 kb) [file 12094_2018_1881_MOESM1_ESM.docx]

Table 1. Clinical questions formulated at the beginning of the consensus process.

|  | [What imaging techniques are currently available for the evaluation of GEP-NETs?](#_¿Qué_criterios_se) |
| --- | --- |
|  | How should GEP-NET patients be stratified depending on the speed of tumor progression? |
|  | Are there any data/findings/imaging biomarkers establishing the patients’ profile? |
|  | When should an active surveillance approach be undertaken in patients with sporadic pancreatic or small intestine NETs? |
|  | What imaging procedures should be performed during the follow-up of patients with GEP-NETs when an ‘active surveillance’ strategy has been selected? |
|  | How often imaging procedures should be performed in patients with GEP-NETs when an active surveillance strategy has been selected? |
|  | How should different levels of hepatic load be defined in order to start systemic treatment of GEP-NETs? |
|  | What imaging technique and what features or technical requirements are the most recommended for evaluating the appearance and/or progression of GEP-NET metastases? |
|  | Does the degree of differentiation/aggressiveness/proliferation affect the imaging techniques used to assess metastases evolution? |
|  | Should the results of a certain imaging test be compared to the immediately previous study or to the one showing the best response to current treatment? |
|  | What imaging frequency should be used to track the evolution of GEP-NET metastases? |
|  | When should we conduct a nuclear medicine test to assess the evolution of metastatic GEP-NETs? |
|  | Can the results of a nuclear medicine test modify the patient evaluation and monitoring strategy? |
|  | In relation to the RECIST criteria, what are the limitations of these criteria to evaluate the treatment response of GEP-NET metastases? |
|  | What criteria are recommended to assess the treatment response with the different treatment options? |
| SSA and chemotherapyTyrosine kinase (TK) and mTOR inhibitors | |
|  | What criteria should be used to assess the treatment response for small and emerging metastases? What criteria should be used to avoid estimating a pseudoprogression to treatment? |
|  | What are the criteria that indicate a need to perform a NM test to evaluate the treatment response of metastatic GEP-NETs? |
